# Study protocol training for life: a stepped wedge cluster randomized trial about emergency obstetric simulation-based training in a low-income country

**DOI:** 10.1186/s12884-020-03050-3

**Published:** 2020-07-28

**Authors:** A. A. C. van Tetering, A. van Meurs, P. Ntuyo, M. B. van der Hout-van der Jagt, L.G.M. Mulders, B. Nolens, I. Namagambe, A. Nakimuli, J. Byamugisha, S. G. Oei

**Affiliations:** 1 Department of Obstetrics and Gynaecology, MUMC+, Maastricht, The Netherlands; 2 Department of Obstetrics and Gynaecology, St. Antonius Ziekenhuis, Nieuwegein, The Netherlands; 3 Department of Obstetrics and Gynaecology, Makerere University and Mulago National Referral Hospital, Kampala, Uganda; 4 Department of Obstetrics and Gynaecology, Máxima Medical Centre, Veldhoven, The Netherlands; 5 Department of Electrical Engineering, Eindhoven University of Technology, Eindhoven, The Netherlands; 6 Department of Obstetrics and Gynaecology, Canisius-Wilhelmina Ziekenhuis, Nijmegen, The Netherlands

**Keywords:** Medical education, Simulation training, Teamwork, Obstetrics, Patient outcome, Low- and middle-income countries

## Abstract

**Background:**

Globally perinatal and maternal mortality rates remain unacceptably high. There is increasing evidence that simulation-based training in obstetric emergencies is associated with improvement in clinical outcomes. However, the results are not entirely consistent. The need for continued research in a wide variety of clinical settings to establish what works, where and why was recommended. The aim of this study is to investigate the effectiveness of an emergency obstetric simulation-based training program with medical technical and teamwork skills on maternal and perinatal mortality in a low-income country.

**Methods:**

A stepped wedge cluster randomized trial will be conducted at the medium to high-risk labour ward at Mulago Hospital, Kampala, Uganda, with an annual delivery rate of over 23,000. The training will be performed using a train-the-trainers model in which training is cascaded down from master trainers to local facilitators (gynaecologists) to learners (senior house officers). Local facilitators will be trained during a four-day train-the-trainers course with an annual repetition. The senior house officers will be naturally divided in seven clusters and randomized for the moment of training. The training consists of a one-day, monodisciplinary, simulation-based training followed by repetition training sessions. Scenarios are based on the main local causes of maternal and neonatal mortality and focus on both medical technical and crew resource management skills. Kirkpatrick’s classification will be used to evaluate the training program. Primary outcome will be the composite of maternal and neonatal mortality ratios. Secondary outcome will comprise course perception, evaluation of the instructional design of the training, knowledge, technical skills, team performance, percentage of ventouse deliveries, percentage of caesarean sections, and a Weighted Adverse Outcome Score.

**Discussion:**

This stepped wedge cluster randomized trial will investigate the effect of a monodisciplinary simulation-based obstetric training in a low-income country, focusing on both medical technical skills and crew resource management skills, on patient outcome at one of the largest labour wards worldwide. We will use a robust study design which will allow us to better understand the training effects, and difficulties in evaluating training programs in low-income countries.

**Trial registration:**

ISRCTN98617255, retrospectively registered July 23, 2018.

## Background

Newborn and maternal healthcare is worldwide open to improvement. In 2000, two out of eight Millennium Development Goals (MDGs) were directed to reduce under-five child mortality and to improve maternal health [1]. Despite the fact that impressive improvements were made in most regions, this progress was not sufficient to meet the defined goals. Globally, 2.6 million babies still die every year in their first month of life and a similar number are stillborn [2]. Additionally, every day about 830 women die from preventable causes related to pregnancy and childbirth around the world [3]. In 2016 world leaders launched a new agenda, which includes a set of 17 Sustainable Development Goals (SDGs) for the next 15 years [4].

One of the targets of the old agenda was to reduce under-five mortality rate by two thirds between 1990 and 2015. The MDG report 2015 showed that this mortality rate has declined by more than half, dropping from 90 to 43 deaths per 1000 live births [5]. Despite these impressive improvements, current trends were not sufficient to meet the target. In addition, a report published in the Lancet in 2014 revealed that the progress in the reduction of neonatal deaths has been much slower than that of children over 4 weeks of age [6]. Approximately a fourth of the neonatal deaths is being attributed to intrapartum-related complications [1, 2, 5]. The highest neonatal mortality rate still occurs in sub-Saharan Africa [5].

The target of MDG five was to achieve universal access to reproductive health services, and to reduce maternal mortality ratio (MMR) by three quarters [5]. While globally MMR results showed a reduction of almost 50%, the risk of maternal death in sub-Saharan Africa remains unacceptably high [5]. It was estimated that in 2015, roughly 303,000 women died during and following pregnancy and childbirth [3]. Almost all of these deaths occurred in low-income countries with a defined maternal mortality ratio of 239 per 100,000 live births versus 12 per 100,000 live births in high income countries [3]. Most of these deaths could have been prevented [3, 5]. A key strategy for improving newborn and maternal care is to ensure that every birth occurs with the assistance of skilled health personnel, meaning a medical doctor, nurse or midwife. Globally, this proportion of deliveries attended by skilled health personnel increased to 71% around 2014 [5]. Yet, this leaves more than one in four deliveries without access to crucial medical care during childbirth.

In September 2015 a new sustainable development agenda was set with 17 goals, each with specific targets to be achieved over the next 15 years [4]. Specific targets are to reduce the global maternal mortality ratio to less than 70 per 100,000 live births, to end preventable deaths of newborns aiming to reduce neonatal mortality to at least as low as 12 per 1000 live births, and to increase health financing and the recruitment, development, training and retention of health workforce in developing countries [4]. We will focus on these targets during this study.

### Uganda

Uganda is one of the low-income countries where newborn and maternal healthcare is open to improvement. The MDG Progress Report on Uganda, delivered in 2013, revealed that the observed trend in the reduction of under-five mortality matched the target trajectory [7]. The perinatal mortality rate in Uganda dropped from 39.3 in 1990 to 22.0 in 2013 [7]. The maternal mortality ratio per 100,000 live births was targeted to be reduced to 131 by 2015. Despite the marked improvements in births assisted by trained health workers and access to care after childbirth, at the end of 2015 the maternal mortality ratio was still 343 compared to 687 in 1990 [8].

### Causes of perinatal and maternal mortality

The majority of perinatal deaths worldwide are caused by preterm birth complications (35%), complications during labour and delivery (24%), and sepsis (15%) [5]. The most important direct causes of maternal mortality in Uganda are postpartum haemorrhage (42%), obstructed or prolonged labour (22%), and complications from abortion (11%) [9]. This was disclosed by a survey of 553 health facilities across Uganda [9]. Another important reason is high blood pressure during pregnancy (pre-eclampsia and eclampsia) [8]. The most common underlying cause of death was inadequate staff numbers [4–7]. Moreover, a national assessment found that only 3% of health facilities expected to offer emergency obstetric care, were able to do so [10]. Nevertheless, the annual Health Sector Performance Report in 2011–2012 found that around half of Government healthcare facilities were providing basic obstetric care or had at least one staff member trained in managing complications in pregnancy and childbirth [7, 10]. Hence, it appears that the management of acute obstetric emergencies is open to improvement.

### Simulation-based training

**Fig. 1:**
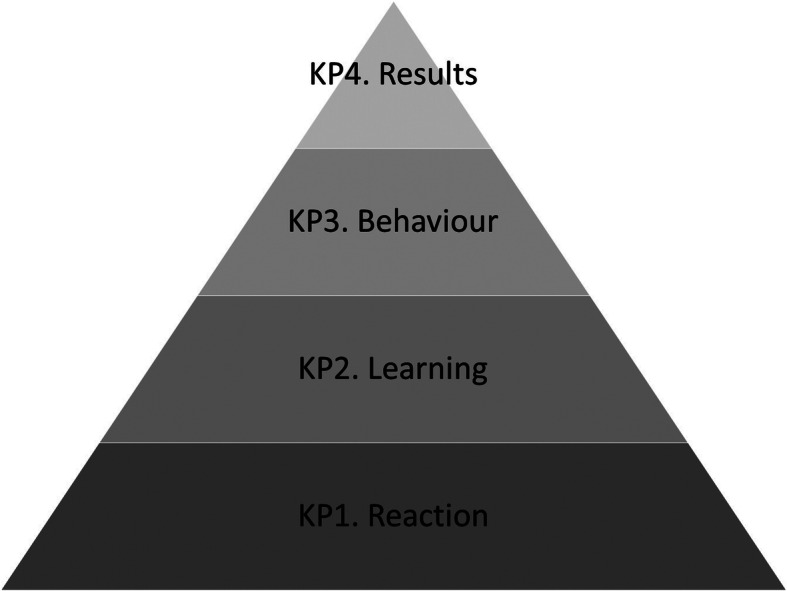
Kirkpatrick’s model for evaluation of training programs. KP: Kirkpatrick level

Simulation-based education (SBE) has increasingly been recognized as a useful and safe educational tool in healthcare over the past decades. To evaluate the effect of a simulation-based education program, Kirkpatrick’s (KP) classification is often used (Fig. 1) [11]. In this classification, four levels are described, starting with trainees’ reaction on the training (KP1), followed by learning (KP2), changes in behaviour (KP3), and finally outcome (patient outcome, reduced cost etc.) (KP4). A systematic review published in 2003 concluded that only a few training programs in acute obstetric emergencies had been described, and even fewer had been evaluated [12]. Since this review, there have been numerous evaluation studies on the effectiveness of simulation training for obstetric emergencies, with increasing evidence that it is associated with improvement in clinical outcomes, mostly related to neonatal outcomes [13–32]. However, the results are not entirely consistent. Training programmes based on local, unit-based and multi-professional training, with appropriate mannequins, and practice-based tools to support best care, were found to be associated with improved clinical outcomes [33]. Yet some training is associated with no improvements, or even deterioration in outcomes. Hence it was recognized that there is a need for continued research in a wide variety of clinical settings to identify which interventions are beneficial [14, 33]. In settings where emergency obstetric simulation-based training is not yet widely established, we propose to use a randomized controlled trial design as most previous studies were set-up as pre- vs. post-interventional design.

## Methods and design

### Aim

The aim of this study is to investigate the effectiveness of a simulation-based emergency obstetric training program on different levels of Kirkpatrick in a low-income country with a stepped wedge cluster randomized trial.

### Design

**Fig. 2:**
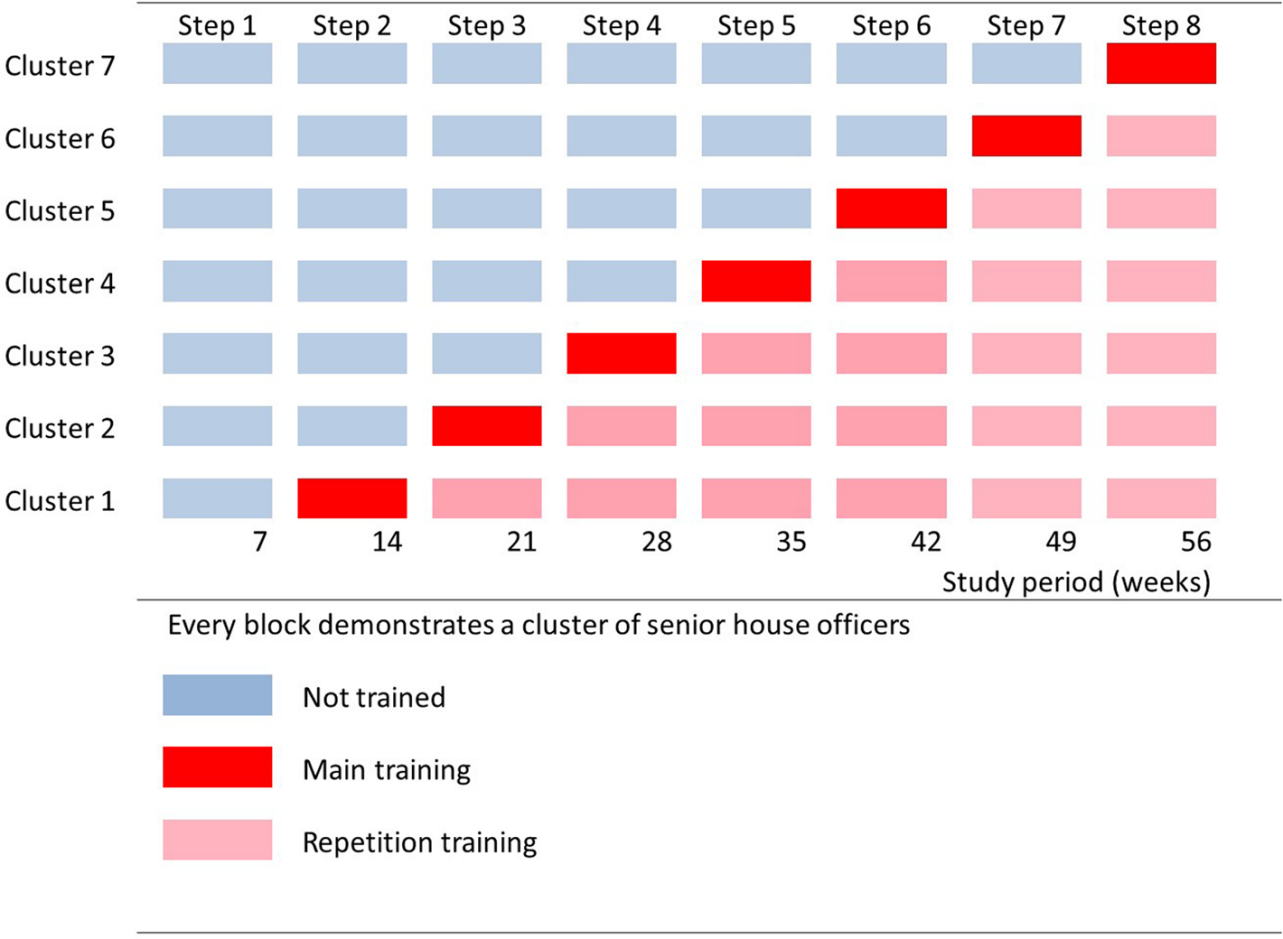
Stepped wedge design with seven clusters and subsequently eight study periods of each 7 weeks

A stepped wedge cluster randomized trial will be conducted at the medium to high-risk labour ward at Mulago Hospital, Kampala, Uganda, with an annual delivery rate of over 23,000. A stepped wedge trial is a cluster-randomized trial in which all study groups (clusters) receive the intervention by a computer-generated random sequential roll-out of the training sessions over time (Fig. 2).

This design has several advantages; all clusters are trained, the effect can be estimated from both within- and between-cluster comparisons, it is possible to control for time effects, and the design is useful when it is preferable to implement the intervention in stages because of logistical, practical or financial constraints [34–37]. However, it may be tough to create fixed clusters of students without contamination during working hours, difficult to plan training sessions during holidays and exam periods, and a challenge to train a new cluster strictly every 7 weeks. Therefore, a prospective pre-post intervention analyses will also be done with 16 months prior to the start of the program and 16 months from the program onwards.

The training will be performed using a train-the-trainers model in which training is cascaded down from master trainer to local facilitators to learners. Local facilitators will be a selection of gynaecologists from Mulago Hospital based on their clinical and teaching experience. They will be selected by the head of department and will train and implement new medical technical skills and crew resource management skills in a hierarchical way (top-down). The gynaecologists will be trained during a 4 day train-the-trainers course including a test day with junior house officers (intern doctors) and annual repetition day. Afterwards, the local facilitators will train the senior house officers (SHOs) in their first-, second- or third-year of their gynaecology training program. The SHOs will be naturally divided in seven clusters of approximately six to nine persons and randomized for the moment of training. The local facilitators will be paid for the training sessions.

The training consists of a one-day, monodisciplinary, simulation-based training followed by repetition training sessions. Scenarios are based on the main local causes of maternal and neonatal mortality and include crew resource management. Primary outcome will be the composite of maternal and neonatal mortality ratios. Secondary outcome will comprise course perception, evaluation of the instructional design of the training, knowledge, technical skills, team performance, percentage of ventouse deliveries, percentage of caesarean sections, and four items of the Weighted Adverse Outcome Score [21].

### Setting and participants

The training will be implemented at Mulago hospital, the national referral hospital in Kampala, Uganda and the teaching Hospital for Makerere University. This hospital serves the population living in Kampala and the surrounding districts. The maternity wards of Mulago Hospital include a low-risk ward and a medium to high-risk ward. Each year, over 31,000 women give birth in Mulago Hospital. Over 23,000 women deliver at the medium to high-risk ward. Only staff from the medium to high-risk ward will be included in this study. The staff consists of 45 gynaecologists, 60 SHOs, and 45 midwives. The SHOs differ in level of education towards their gynaecologist training (first-, second-, and third-year SHOs). During a 24-h shift one gynaecologist is on-call duty. During daytime, six SHOs, and eight midwives provide obstetric care while four SHOs and six midwives are available during night.

The study concerns a simulation-based training program focusing on medical technical skills and crew resource management in emergency obstetric scenarios in a low-income setting. Except for vacuum extraction and neonatal resuscitation training, no other simulation-based training was done before. The local facilitators will train all first-, second- and third-year SHOs since they have a central and coordinating role in providing emergency obstetric care on the labour ward in this hospital. With over 23,000 women giving birth at the ward in 1 year, and only gynaecologist on-call duty during a 24-h shift, midwives ask the SHOs to handle in case of an emergency obstetric situation. Before the SHOs start with their three-year training to become a gynaecologist, they have worked already in the hospital as an intern during their training to become a doctor. During the period as an intern, they are already responsible for triage of patients and learn how to perform a caesarean section. After their internship, students got selected to become a resident in obstetrics and gynaecology. During a shift, the SHOs are working together in teams of first-, second-, and third-year students. They need to call and help each other during emergency obstetric situations.

The training program consists of a full-day simulation-based training and half-day repetition training sessions. To be included, SHOs must work at the medium to high-risk maternity ward of the Mulago hospital. Written informed consent to participants in this study have to be obtained at the beginning of the first training day.

### Trial interventions

#### Training equipment

Full body simulators (Noelle® and Pedi® Blue neonate, Gaumard) will be used for all training sessions. Communication with Noelle® will be done by an actor, usually a non-local doctor who is not involved in the training day. Other training materials (e.g. disposables, balloon ventilators) will be obtained from Mulago Hospital labour ward and reused as much as possible. Course content (e.g. syllabus, instructor manual, slides, observation forms) will be developed in cooperation with staff members of the obstetrics and gynaecology department in Mulago Hospital and Medsim, a medical simulation centre in Eindhoven, The Netherlands. All materials will be written in English. The instructional design features described by Issenberg et al. and Cook et al. will be used to design the training program [38, 39].

#### Train-the-trainers course

All training sessions will take place at the skills lab of the Makerere University College of Health Sciences, situated at Mulago hospital. The first step will be the train-the-trainers course for the local facilitators provided by a Dutch team, consisting of two obstetricians, a communication expert and a simulation technician. They are all certified simulation educators. The train-the-trainers course will take 4 days. During the first day, principles of simulation-based education (e.g. learning theories, crew resource management, debriefing techniques) and the training program for the SHOs will be discussed and registered. Additionally, local protocols for obstetric emergencies will be restructured during the first day and the local trainers will be introduced to the full body simulators. From the second day on, the local facilitators will start practicing during obstetric simulated scenarios on each other. This includes preparing, leading and debriefing a simulated scenario, including technical set-up (simulators, audio-video equipment, presentation equipment). On the last day the trainers will educate junior house officers (intern doctors) in simulation-based obstetric training as a final test. The Dutch team of master trainers will provide the local trainers feedback. Afterwards, the local trainers will facilitate simulation-based training sessions for all SHOs without supervision of the master trainers in the above mentioned training scheme. Each training session will be organised by two local trainers (main and repetition training). Finally, the master trainers will provide an annual one-day train-the-trainers repetition course.

#### Main training sessions

**Fig. 3:**
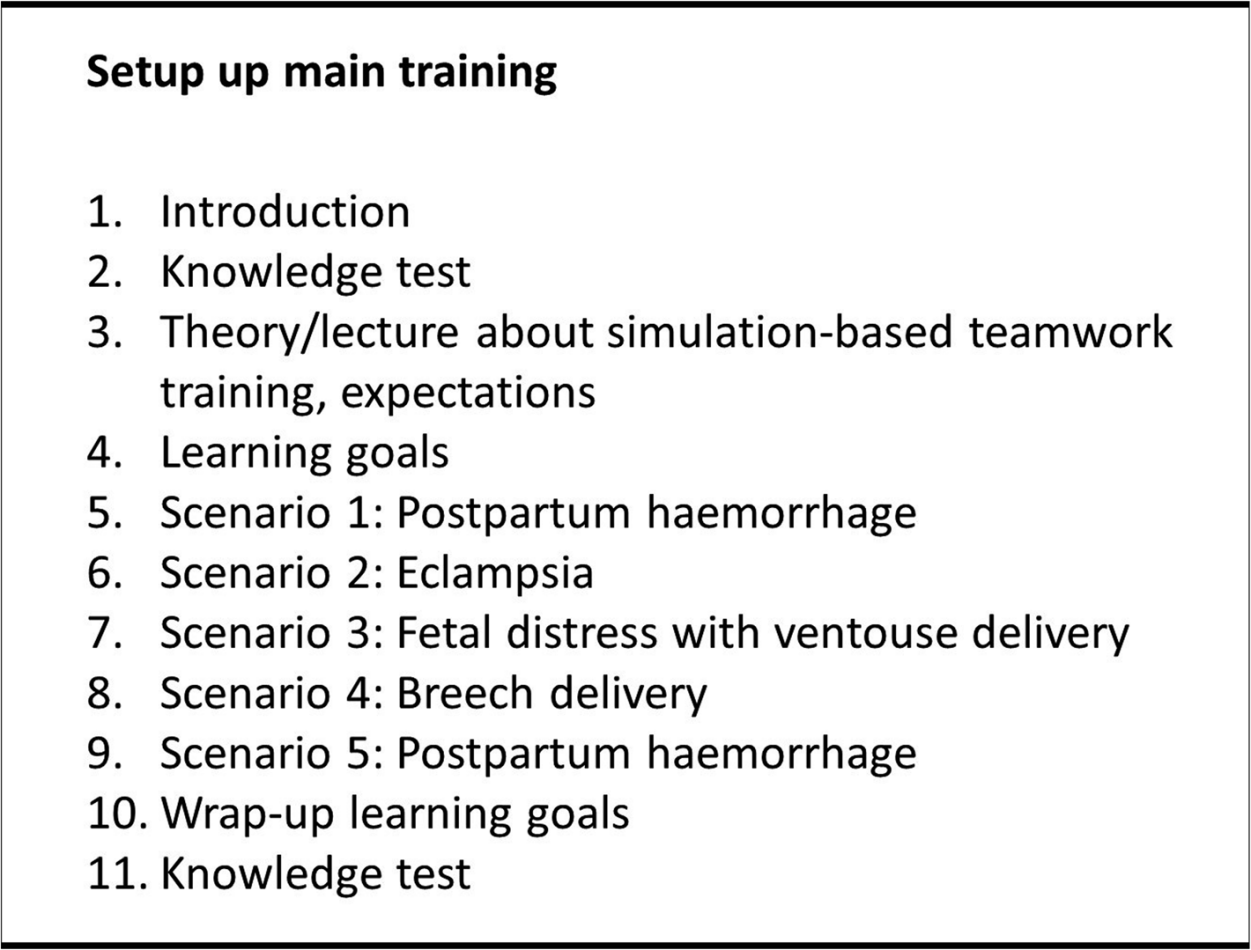
Set up of the main training

The main training of the SHOs will comprise a one-day (8-h), simulation-based, obstetric training, focusing on 50% medical technical skills and 50% crew resource management skills (i.e. teamwork skills) (Fig. 3).

There are seven groups with SHOs, each group consists of six to nine SHOs. The training scenarios will be postpartum haemorrhage, eclampsia, fetal distress with a vacuum-assisted vaginal delivery and resuscitation of the newborn, breech delivery, and a repetition scenario of postpartum haemorrhage. These scenarios relate to local leading causes of perinatal and maternal mortality and obstetric healthcare problems. Crew resource management skills such as speak up, leadership, situational awareness, and decision-making will be integrated in every scenario with increasing difficulty levels. Learning goals and learning objectives will be defined for the scenarios in collaboration with the local trainers.

The main training will start with a general introduction and a knowledge test. Afterwards, the concept of simulation-based training will be explained and the SHOs will set their individual learning goals. Subsequently, the five scenarios will be covered. Every scenario will start with a short introduction of the scenario, followed with performing the scenarios by two to three trainees. The trainers and remaining SHOs will observe the trainees via synchronised video broadcast. Every trainee will participate in at least two scenarios. After each scenario a debriefing by means of the video recordings will be provided by the trainers. The video will be used to show what was done/not done. After this, the students will reflect upon their learning experiences. The debriefing will contains three different phases; reaction, analysis and take home phase. The instructors will provide feedback on both medical technical skills and crew resource management skills. When all five scenarios are completed, the predefined learning goals will be evaluated. Finally, all SHOs will undergo the same knowledge test and they will be asked to fill in an evaluation questionnaire on course perception.

#### Repetition training sessions

After the main training, SHOs will be invited to take part in repetition training sessions. Each repetition training session will comprise half a day. During a repetition training, one clinical scenario will be executed (and repeated). New scenarios will be designed for these sessions based on the same emergency obstetric situations in the main training. However, expectations for the level of performance will be raised and some extra elements such as hand hygiene will be added to keep it challenging. Each repetition training will start with an introduction, in which learning goals will be defined. At the end of every training, learning goals will be evaluated and summarized.

### Hypothesis

Simulation-based obstetric training in a low-income country will decrease the composite of maternal and perinatal mortality ratios. Questions to be answered:

1. To what extent is emergency obstetric simulation-based training acceptable and feasible for local facilitators and trainees in a low-income country? (KP 1)
2. Does emergency obstetric simulation-based training provided by local facilitators in a low-income country improve knowledge, skills, and team performance? (KP 2)
3. Does emergency obstetric simulation-based training provided by local facilitators in a low-income country increase the percentage of deliveries by vacuum extraction and decrease the percentage of caesarean sections? (KP 3)
4. Does emergency obstetric simulation-based training provided by local facilitators in a low- income country decrease four items of the Weighted Adverse Outcome Score? (maternal death, intrapartum or perinatal death, uterine rupture, Apgar score less than 7 at 5 min) (KP 4)
5. Does emergency obstetric simulation-based training provided by local facilitators in a low- income country decrease the composite of maternal and perinatal mortality ratios, the separate ratios, and the ratio of maternal and perinatal mortality per total number of deliveries? (KP 4)

### Primary outcome

The primary outcome of this study will be the combined mortality proportion (CMP). This will concern a composite mortality rate, including maternal and perinatal mortality. Maternal mortality ratio (MMR) is defined as the number of maternal deaths per 100,000 live births [40]. Perinatal mortality ratio (PMR) is defined as the number of stillbirths and deaths in the first week of life per 1000 live births. In Mulago hospital only deliveries with a gestational age of 28 weeks or more or a birth weight of more than one kilogram are registered. These deliveries will be included in the analysis.

Expressed as proportions, the combined mortality proportion (CMP) holds: CMP = MMR/100,000 + PMR/1000. Maternal mortality and perinatal mortality will be prospectively registered using the patient registration books in Mulago Hospital. Data extraction from these registration books will be without identification of the subjects.

### Secondary outcomes

To evaluate the perception of the trainees of the training program, we will use a 42-item questionnaire about ten instructional design features of the training program including feedback, repetition, curriculum integration, difficulty range, learning strategies, clinical variation, controlled environment, individualization, defined outcomes, and simulator validity (the ID-SIM) [41]. The data will be treated as ordinal data at the item level. Suggestions for improvement can be made in an open remark.

A 30-item multiple choice knowledge test about technical and non-technical skills was development and evaluated by gynaecologists from Mulago Hospital and the Netherlands. SHOs will be asked to fill in the questionnaire at the onset and end of the full-day main training. Mean values of the knowledge tests will be compared.

Clinical performance in the simulated postpartum haemorrhage scenarios will be assessed by three independent Dutch simulation instructors through reviewing the videotaped training sessions. The assessors will be blinded for the day of training and whether the scenario was the first or the last of the day. A skills checklist based on literature and clinical experience will be used [42]. The mean score of the clinical performance of the first and last scenario of the SHOs’ main training will be compared.

Team performance will also be assessed by three independent Dutch simulation instructors through reviewing the videotaped training sessions. The Clinical Teamwork Scale (CTS) will be used [43]. The CTS contains questions about communication, situational awareness, decision-making, role responsibility, and patient friendliness. The mean score of the clinical performance of the first and last scenario of the main training will be compared. The assessors will be blinded for the day of training and whether the scenario was the first or the last of the day.

The percentage of deliveries by vacuum extraction and caesarean sections will be prospectively collected from Mulago Hospital’s patient registration book.

**Fig. 4:**
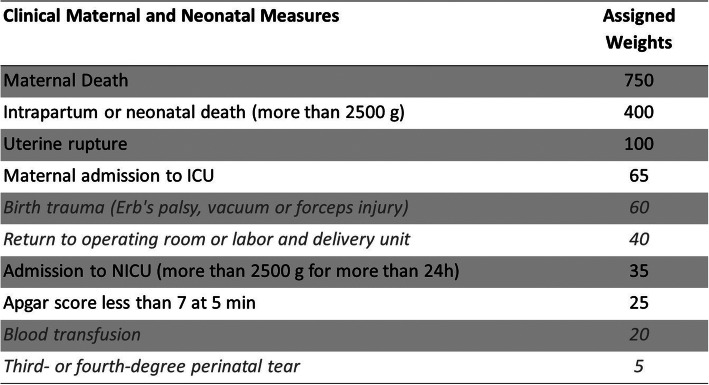
Weighted Adverse Outcome Score (WAOS). ICU, intensive care unit; NICU, neonatal intensive care unit [21].

The Weighted Adverse Outcome Score (WAOS) is defined as the total weighted score of each adverse outcome divided by the total number of deliveries (Fig. 4) [44]. Because of registration difficulties, only four out of 10 index measures (maternal death, intrapartum or perinatal death, uterine rupture, Apgar score less than 7 after 5 min) will be assessed.

Maternal and perinatal mortality ratios will also separately be evaluated, because these ratios are not independent in the combined mortality proportion. Finally, the ratio of maternal and perinatal mortality per total number of deliveries will be analysed.

### Sample size calculation

Power calculation was carried out as described in both Hussey et al. and Woertman et al. [35–37] First the sample size calculation for a standard randomized clinical trial (RCT) was calculated. To show a reduction in CMP of 20% with an alpha of 0.05 and a power of 80%, a total of 6398 deliveries will be needed for a simple RCT design. The design effect was calculated assuming an intracluster correlation (ICC) of 0.05, a cluster size of 3343 deliveries per year, and seven clusters. Considering the design effect, we will need 2367 deliveries per measurement period. To achieve this number at least 5 weeks for each period will be needed. However, to obtain logistical possibilities, the duration of each step will be 7 weeks with a total study duration of 56 weeks. Statistical significance will be accepted at a two-sided *p*-value < 0.05.

### Statistical analysis

All data will be collected, secured and stored in Mulago hospital. Access to the final trial dataset will be limited to persons who have to perform statistical analyses and to interpret results. The statistical analysis of the stepped wedge design will be analysed in different steps. First of all, differences in patient characteristics (age, parity, gestational age, single/multiple pregnancy, neonatal gender, birth weight) across clusters will be investigated with Kruskal-Wallis for numerical data and with exact chi-square statistics for categorical data. The characteristics that seem to be different across clusters (*p* < 0.05) will enter into the generalized linear mixed effects model (GLMM) for estimation of treatment effect. Here the outcome is the event (i.e. composite mortality rate, including maternal and perinatal mortality) on the individual and we will use a logit link function to model the probability of the event. In the logit scale, the cluster indicator will serve as random effect on the intercept, the selected patient characteristics will enter this model as linear predictors, the period of the stepped wedge is treated as categorical effect, as well as the treatment effect.

In mathematical terms the model can be formulated as follows:

$$ \mathrm{logit}\left[P\left({Y}_{ij}=1|{Z}_i,{\boldsymbol{P}}_{ij},{\boldsymbol{X}}_{ij},{T}_{ij}\right)\right]=\mu +{Z}_i+\sum \limits_{r=1}^R{\alpha}_r{P}_{ij r}+\sum \limits_{s=1}^S{\beta}_s{X}_{ij s}+\gamma {T}_{ij} $$

with the logit function given by logit(*x*) = log *x* − log(1 − *x*) and

*Y*_*ij*_: the binary outcome on patient *j* in cluster *i*,

*Z*_*i*_: the random effect of cluster *i*, assumed to be normally distributed ${Z}_i\sim N\left(0,{\sigma}_C^2\right)$, and with ${\sigma}_C^2$ the between-cluster variance,

***P***_*ij*_: a vector of indicators ***P***_*ij*_ = (*P*_*ij*1_, *P*_*ij*2_, …, *P*_*ijR*_)^*T*^ for patient *j* in cluster *i* that indicate in which period the observation is taken, with ${\sum}_{r=1}^R{P}_{ijr}=1$ and *R* the number of periods,

***X***_*ij*_: a vector of confounders ***X***_*ij*_ = (*X*_*ij*1_, *X*_*ij*2_, …, *X*_*ijS*_)^*T*^ for patient *j* in cluster *i*, and *S* the number of covariates.

*T*_*ij*_: the treatment indicator for patient *j* in cluster *i*.

*μ*: the overall intercept,

*α*_*r*_: the effect of period *r*

*β*_*s*_: the effect of confounder *X*_*ijs*_

*γ*: the effect of treatment [45].

### Ethical considerations

Ethical approval was obtained from both the Mulago Research and Ethics Committee (Protocol MREC: 674), and the Uganda National Council for Science and Technology (UNCST, SS 3927). Written informed consent to participants in this study was obtained at the beginning of the first training day.

## Discussion

In the last decades numerous evaluation studies on the effectiveness of simulation training in obstetric emergencies have been published, with increasing evidence that it is associated with improvement in clinical outcomes [13–32]. However, the results are not entirely consistent. The need for continued research in a wide variety of clinical settings to establish what works, where and why was recommended [14, 33]. Therefore we propose to use a randomized controlled trial design in settings where emergency obstetric simulation-based training is not yet widely established.

This stepped wedge cluster randomized trial will investigate the effect of a monodisciplinary simulation-based obstetric training focusing on both medical technical skills and crew resource management skills in a low-income country, on patient outcome at one of the largest labour wards worldwide. In this study, also other non-patient outcome measures will be included. We hypothesize that simulation-based obstetric training in a low-income country is acceptable and feasible for trainers and trainees, improves knowledge, skills, team performance and professional practice, and improves maternal and neonatal morbidity/mortality rates. Different from previous studies, we will use a robust study design which will allow us to better understand the training effects.

A limitation of this most accurate study design in one hospital is the need of fixed teams without contamination during working hours. Due to organizational constraints, such as exam periods and cluster inconsistency, this design may be hard to implement in a low-income country. Therefore pre-post analyses will be performed as well. Additionally, it was logistical impossible to create multidisciplinary fixed teams including midwives, junior house officers, SHOs, gynaecologists, paediatricians, and anaesthesiologist. Team training was set up monodisciplinary, with only first-, second-, and third-year SHOs. This group was chosen as they have a central and coordinating role in providing emergency obstetric care in this hospital, and they have to work in teams during their shift. However, training all obstetric team member would possibly contribute more to reduce maternal and neonatal morbidity and mortality. Another limitation is the selection of only gynaecologists to become local facilitators. They were selected by the head op department based on their clinical and teaching experience. Future studies should include other obstetric team members as facilitators and should include evaluation of personal suitability.

The primary outcome will be a composite of the maternal mortality ratio (MMR) and the perinatal mortality ratio (PMR). While there is a clear definition of the perinatal mortality ratio, issues with routine antenatal care visits and administration difficulties will give uncertainties in gestational age and moment of death. This will maybe influence data collection and interpretation. Improvement of data administration seems to be an important worldwide issue.

The current project fits well within the formulated Sustainable Development Goals agenda to ensure healthy lives and to promote wellbeing for newborns and mothers by specifically increasing the development, training and retention of health workforce in developing countries [4]. If this training program appears to be effective, this training should be implemented in the curriculum of the gynaecology training program in comparable low-income settings.

## Supplementary information

**Additional file 1.** Knowledge test. 30-item multiple choice knowledge test about technical and non-technical skills.

**Additional file 2.** Skills checklist. Skills checklist of postpartum haemorrhage based on literature and clinical experience.

## Data Availability

The datasets which will be used and analysed during the current study will be available from the corresponding author on reasonable request.

## Abbreviations

MDG: Millennium development goals
SHO: Senior house officer
CRM: Crew resource management
CTS: Clinical teamwork scale
Medsim: Medical education and simulation centre Eindhoven
CMP: Combined mortality proportion
MMR: Maternal mortality ratio
PMR: Perinatal mortality ratio
WAOS: Weighted adverse outcome score
RCT: Randomized clinical trial
