# Supplementary material for: Study protocol training for life: a stepped wedge cluster randomized trial about emergency obstetric simulation-based training in a low-income country
Source: BMC Pregnancy Childbirth. 2020 Jul 28;20:429. doi: 10.1186/s12884-020-03050-3 (PMC7388496; doi:10.1186/s12884-020-03050-3)
Supplement: Supplementary file 2 — Additional file 2. Skills checklist. Skills checklist of postpartum haemorrhage based on literature and clinical experience. [file 12884_2020_3050_MOESM2_ESM.docx]

**Skills checklist**

**Assessor 1**

**Video no.: _____________________**

|  | **Yes** | **No** | **N/A** |
| --- | --- | --- | --- |
| **Help**  1. Ask for more assistance (midwife, SHO, gynaecologist) |  |  |  |
| **Airway, breathing, circulation**  2. Fluid (2 IV lines) N/S or Ringers lactate is given |  |  |  |
| 3. O2 15L/min is provided |  |  |  |
| 4. Blood sample is taken for investigation |  |  |  |
| 5. If in shock: aorta compression is given and legs are elevated |  |  |  |
| 6. Left lateral tilt: patient is positioned on left side |  |  |  |
| 7. Blood pressure is assessed |  |  |  |
| 8. Heart rate is assessed |  |  |  |
| 9. Amount of blood loss is estimated |  |  |  |
| **Establish cause**  10. Check if placenta is complete (if not: manual removal (pethidine)) |  |  |  |
| 11. Insert catheter to empty bladder |  |  |  |
| 12. Check for tears (if so: repair) |  |  |  |
| 13. If coagulopathy: FFP, platelets or fresh blood |  |  |  |
| **Massage uterus**  15. Uterus massage is given |  |  |  |
| 16. Clots are expelled |  |  |  |
| 17. Bimanual compression is given |  |  |  |
| **Oxytocin (drugs)**  18. Pitocin is given |  |  |  |
| 19. Ergometrin is given |  |  |  |
| 20. Misoprostol is given |  |  |  |
| 21. Tranexamic acid is give |  |  |  |

|  | **Yes** | **No** | **N/A** |
| --- | --- | --- | --- |
| **Tamponade**  22. Bakri balloon |  |  |  |
| **Shift to theatre**  23. Notify OR-team and anaesthesiologist |  |  |  |
| 24. Go to the operating theatre |  |  |  |

**Additional notes:** _______________________________________________________________________________________________________________

**_______________________________________________________________________________________________________________**

**_______________________________________________________________________________________________________________**
